# Visualizing enteric nervous system activity through dye-free dynamic full-field optical coherence tomography

**DOI:** 10.1038/s42003-023-04593-9

**Published:** 2023-03-02

**Authors:** Tony Durand, Perrine Paul-Gilloteaux, Michalina Gora, Lara Laboudie, Emmanuel Coron, Isabelle Neveu, Michel Neunlist, Philippe Naveilhan

**Affiliations:** 1 Nantes Université, CHU Nantes, Inserm, TENS, The Enteric Nervous System in Gut and Brain Diseases, IMAD, Nantes, France; 2 Nantes Université, CNRS, INSERM, l’institut du thorax, F-44000 Nantes, France; 3 Nantes Université, CHU Nantes, Inserm, CNRS, SFR Santé, Inserm UMS 016, CNRS UAR 3556, F-44000 Nantes, France; 4 Wyss Center for Bio and Neuroengineering, Campus Biotech, Geneva, Switzerland; 5 ICube Laboratory, CNRS, Strasbourg University, Strasbourg, France; 6 Department of Gastroenterology and Hepatology, University Hospital of Geneva (HUG), rue Gabrielle Perret-Gentil 4, 1211, Genève, 1205 Switzerland

**Keywords:** Optical imaging, Neurophysiology

## Abstract

Major advances have been achieved in imaging technologies but most methodological approaches currently used to study the enteric neuronal functions rely on exogenous contrast dyes that can interfere with cellular functions or survival. In the present paper, we investigated whether full-field optical coherence tomography (FFOCT), could be used to visualize and analyze the cells of the enteric nervous system. Experimental work on whole-mount preparations of unfixed mouse colons showed that FFOCT enables the visualization of the myenteric plexus network whereas dynamic FFOCT enables to visualize and identify in situ individual cells in the myenteric ganglia. Analyzes also showed that dynamic FFOCT signal could be modified by external stimuli such veratridine or changes in osmolarity. These data suggest that dynamic FFOCT could be of great interest to detect changes in the functions of enteric neurons and glia in normal and disease conditions.

## Abstract (web summary)

Full-field optical coherence tomography (FFOCT) can be used for the visualization of the myenteric plexus network and individual myenteric ganglia cells in situ, showing that FFOCT is a suitable method to image enteric nervous system activity.

## Introduction

The enteric nervous system (ENS) is an integrated neuronal network present within the gastro-intestinal (GI) tract. Composed of neurons and enteric glial cells (EGC), the ENS regulates key GI functions encompassing motility, mucosal secretion, cell proliferation, tissue repair, but also immune functions^1^. Defects in the ENS organization and functions contribute to various GI dysfunctions observed in a wide spectrum of digestive and extra-digestive disorders^2^.

Over the past years, microscopic imaging techniques have been developed to better understand ENS functions under normal and abnormal conditions. For instance, progress have been made by combining high-resolution microscopic techniques with fluorimetric probes such as calcium-sensitive or voltage-sensitive dyes^3^. Insight into ENS organization and vasculature has also been provided by technologies like light-sheet microscopy and optical projection tomography, which allow the analysis of sections of a few square centimeters of excised digestive tissue^4,5^. However, these technical approaches can be performed only ex vivo as they require tissue clearing and immunohistochemical staining. In vivo visualization of the human ENS was achieved using probe confocal endomicroscopy (pCLE) but as the resolution and penetration depth were too low, endoscopic submucosal dissection^6^ or cresyl violet staining^6^ was necessary to obtain a clear image of the ENS^7^. In this context, novel technologies providing high spatial resolution and dye-free dynamic imaging of the ENS is of great interest.

To this regard, the optical coherence tomography (OCT) can be very useful since it enables the imaging of tissue internal structures by interferometrical analysis of backscattered and reflected light^8^. This high-speed and noninvasive technology relies on inherent tissue contrast to create volumetric reconstruction of the tissue. This imaging strategy is most widely used to diagnose disease in the human eye^9^, but when combined with a fiber optic probe, the OCT could acquire images in the cardiovascular, respiratory, or digestive system^10^. In the gut, endoscopic OCT has been typically used for the diagnosis of Barrett’s esophagus^11^ and preliminary data was obtained for other indications like inflammatory bowel diseases and colorectal cancer^12,13^. In vivo endomicroscopic OCT with a typical resolution of tens of microns^14^ has a too low resolution to visualize the ENS, but higher resolution can be obtained with two implementations of this technique: FFOCT^15,16^ and high-resolution SD-OCT^17–19^. In this paper we present results obtained with the FFOCT, which acquires 2D en face images using high numerical aperture objectives and visible light. It allows the generation of images with micrometric and subcellular resolution and volumetric data can also be collected by changing the acquisition depth of the OCT en face image. Its application has been mostly explored for intra-procedural tumor margin detection in various organs^20^, including the brain^21^. It has also been reported for imaging the eye structures^22,23^. Despite some early efforts^24^, the development of an endoscopic version of the FFOCT was so far not achieved. In the ex vivo samples of the gut, FFOCT has demonstrated its ability to image the myenteric plexus in the GI tract of mouse and human^25^. However, this imaging method does not visualize the neurons and the glial cells in the enteric ganglia, in part due to the strong backscattering signals produced by surrounding tissues such as muscle cells or by the ENS and the presence of a speckle noise. A new tool called dynamic FFOCT (D-FFOCT) have greatly improved the cellular contrast in complex tissue, by analyzing the temporal fluctuations of backscattered light from tissue and cells^26^. For temporal analysis of micromotion of cell constituents, which is at the bases of the intrinsic contrast of the D-FFOCT, the OCT signals are collected over time from the same imaging plane, which in turn also reduces add on noise and improves image quality. The dynamic contrast was also implemented in the high-resolution SD-OCT technology, which provides cross-sectional images of the tissue, in comparison to en face imaging plane of the D-FFOCT^27^. Micro-OCT with dynamic contrast was used for visualization of micro-anatomy of airways^28^, cervix, and esophagus^29^. Recent efforts are focused on further improving of an acquisition speed and image quality of D-FFOCT and micro-OCT with dynamic contrast to enable in vivo imaging^30–32^. At the same time, micro-OCT technology has been successfully translated to an endoscopic design^33,34^. Preliminary design of micro-OCT endoscope with the dynamic contrast was presented^35^ but the challenge of tissue stabilization during long data acquisition necessary to extract the dynamic contrast remains as a major limitation. The cellular mechanisms underlying temporal fluctuations of the OCT signals also remain unknown but changes in cellular metabolic activity might be partially responsible as the inhibition of mitochondrial activity using rotenone, or of glycolysis using 2-deoxy-D-glucose significantly reduced the OCT signal intensity^26^. Interestingly, individual epithelial or immune cells which could not be identified individually by FFOCT, could be visualized by D-FFOCT^36^. This is encouraging but to date, nothing is known about the ability of D-FFOCT to image the ENS or the biological mechanisms underlying the D-FFOCT signals from the ENS cells.

In the present study, excised colonic segments from adult mice were analyzed by FFOCT and D-FFOCT to demonstrate the ability of this technology to image neurons and glial cells in the myenteric plexus, and to measure changes in D-FFOCT signals linked to neuronal electrophysiological activity.

## Results

### FFOCT imaging enables the identification of the myenteric plexus

**Fig. 1:**
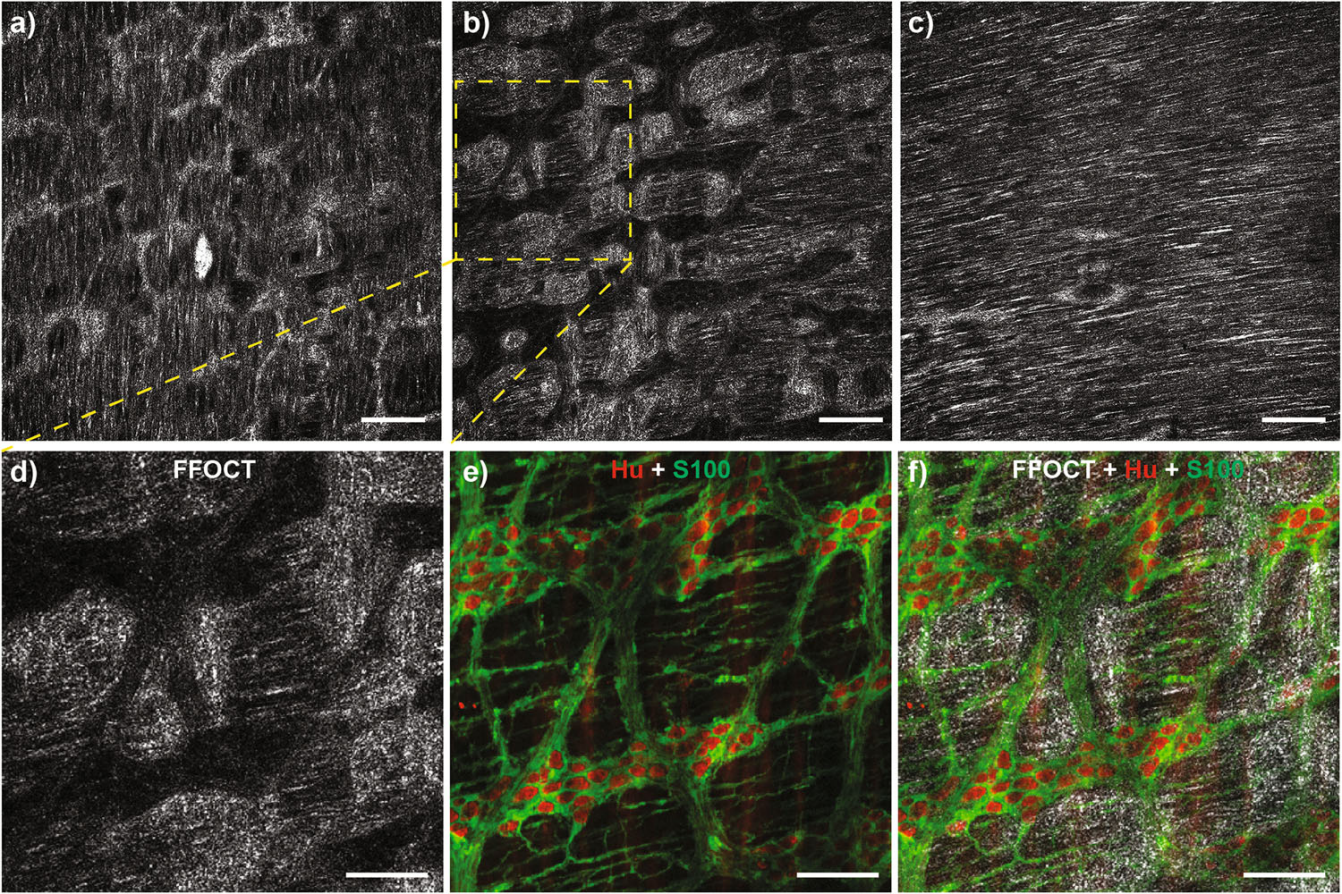
FFOCT identification of the myenteric plexus in the mice colon. **a**–**c** The mice colon was analyzed by FFOCT at the level of **a** longitudinal muscle, **b** myenteric plexus, and **c** circular muscle. Selected area of the myenteric plexus was visualized by **d** FFOCT and by **e** apotome after the immunolabelling of neurons (Hu in red) and enteric glial cells (S100b in green). The merged image (**f**) confirms that the dark areas observed by FFOCT correspond to ganglia and interganglionic fiber tracts. Scale bar: **a**–**c** 200 µm; **d**–**f** 100 µm.

To determine whether FFOCT can be used to identify myenteric ganglia without any dye-labeling, whole-mount preparations of distal colon segments from mice were pinned on Sylgard support and imaged using FFOCT. The images were acquired at consecutive depths of 0.5 µm starting at the level of the external serosa. The longitudinal (Fig. 1a) and circular muscle (Fig. 1c) layers were clearly identified as their fibers are perpendicular to one another. Interestingly, the ten to twelve FFOCT images recorded in between the two muscle layers showed dark network delineated by refractory regions (Fig. 1b). To confirm that these dark structures corresponded to the myenteric plexus, tissue samples previously imaged by FFOCT were immunostained using antibodies against Hu and S100β to identify neurons and enteric glial cells, respectively (Fig. 1d–f). Using these combined approaches, we were able to show that FFOCT can be used to identify structures formed by ganglia and interganglionic fiber tracts since the dark network observed in FFOCT superimposed with the Hu/S100β staining (Fig. 1f). However, individual neurons and/or glial cells present within the myenteric plexus could not be identified using FFOCT.

### D-FFOCT analysis enables the visualization of individual cells within the myenteric ganglia

In D-FFOCT, the images of selected area are acquired over time allowing analysis of the movements in each voxel, and thus, providing additional contrast that enables resolving cells and nucleus. Using this approach, we were previously able to identify individual epithelial cells along the human gut^36^. Here, we have analyzed the tissue samples by D-FFOCT to determine whether this technique can be used to identify individual cells within the myenteric ganglia.

**Fig. 2:**
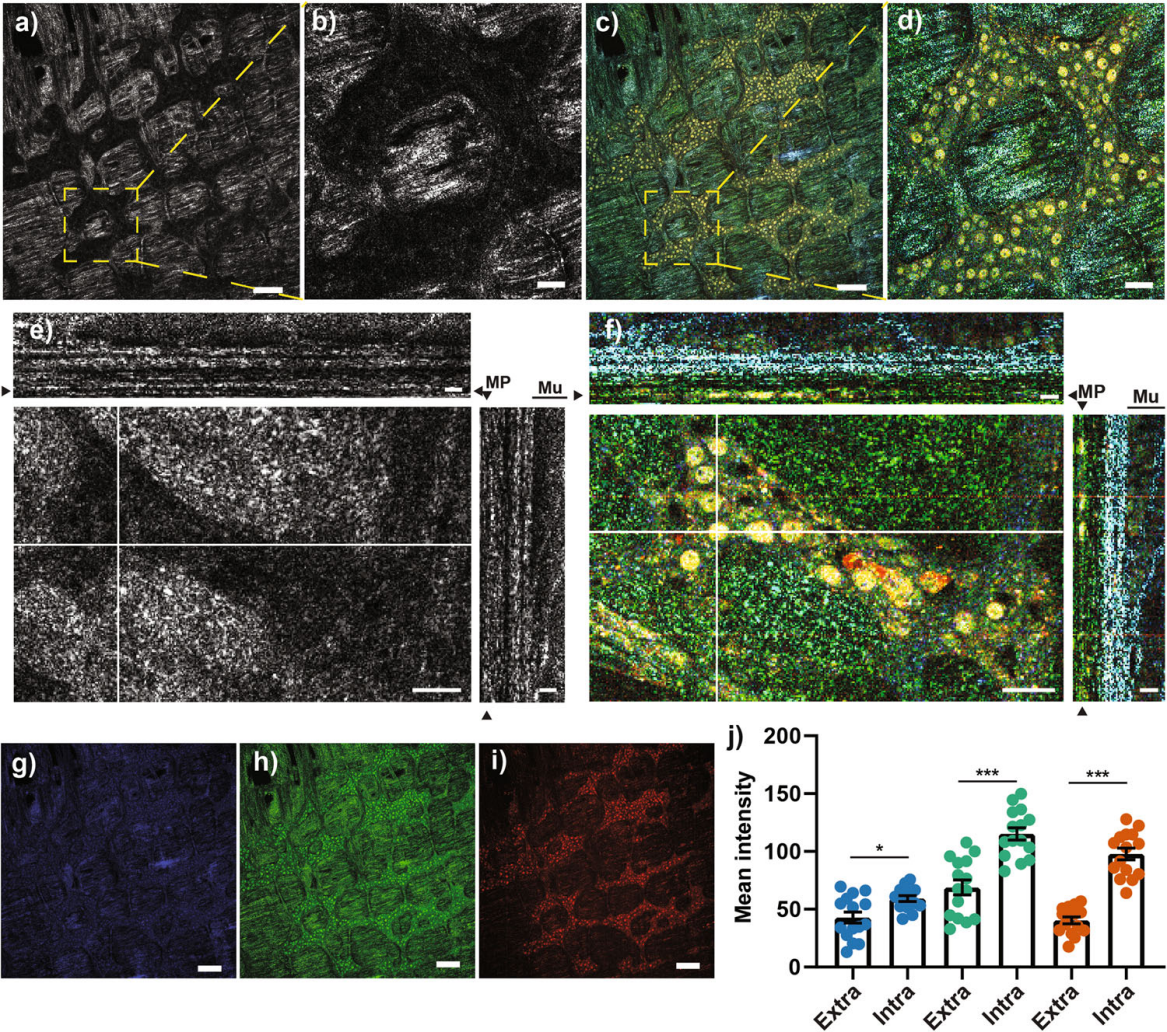
Comparison of FFOCT and D-FFOCT imaging of the myenteric plexus in the mouse colon. **a**–**d** Micrograph of the myenteric plexus region in FFOCT (**a**, **b**) or D-FFOCT (**c**, **d**). **b** and **d** represent an enlargement of the dotted area in (**a**) and (**c**), respectively. **e**, **f** Orthogonal view of different planes (x/y; x/z and y/z) of FFOCT (**e**) and D-FFOCT (**f**) z-stack images (60 images). Arrows head point out the position of “en face image” in the orthogonal view. **g**–**i** The D-FFOCT micrograph in (**c**) is a RGB-coded image in which the blue channel (**g**) represents the low frequencies [0–0.6 hertz], the green channel (**h**) corresponds to medium frequencies [0.6–5.4 hertz] and the red channel (**i**) to high frequencies [5.4–25 hertz]. **j** The mean intensity of low (blue), medium (bluish green), or high (vermillion) frequencies was determined in intra- and extra-ganglionic regions of the myenteric plexus. Three animals were used to determine the mean intensity +/− SEM and five independent regions were analyzed per animal. Statistic: two-tailed Mann and Whitney t-test. *p < 0.05; ***p < 0.001. Scale bar: **a**, **c**, **g**–**i**, 125 µm; **b**, **d**, 30 µm; **e**–**f**, x/y 30 µm, x/z and y/z 12 µm. MP myenteric plexus, Mu mucosa.

As illustrated in Fig. 2, comparison of the FFOCT and D-FFOCT images clearly showed structures which were only detected by the dynamic mode (Fig. 2a–f). Some of these structures were probably individual nucleus as indicated by their morphology and their localization in the ganglia. D-FFOCT images generated by the Light-CT scanner are pseudo-colored RGB images where each color channel is built on the Fourier-domain analysis of the micromotions integrated on three different frequency ranges. The low [0–0.6 Hz], medium [0.6–5.4 Hz] and high [5.4–25 Hz] frequencies were color-coded into blue, green, and red, respectively (Fig. 2g–i). To improve the contrast, and visualize and quantify functional information, the RGB images were split into monochrome images corresponding to their three color channels. Using this approach, we were able to discriminate between ganglionic and extra-ganglionic structures due to differences in signal intensity and amplitude within the tissue. First, amplitude of all frequency signals was significantly higher in ganglionic structures as compared to extra-ganglionic structures (Fig. 2j). Furthermore, in ganglionic structures, the amplitude of high-frequency signals was higher compared to the low-frequency signals. This was not the case in extra-ganglionic structures. Finally, in both structures, the amplitude of medium-frequency signals was higher as compared to low and high-frequency ones.

### D-FFOCT enables the visualization of individual neurons and glial cells within the myenteric plexus

**Fig. 3:**
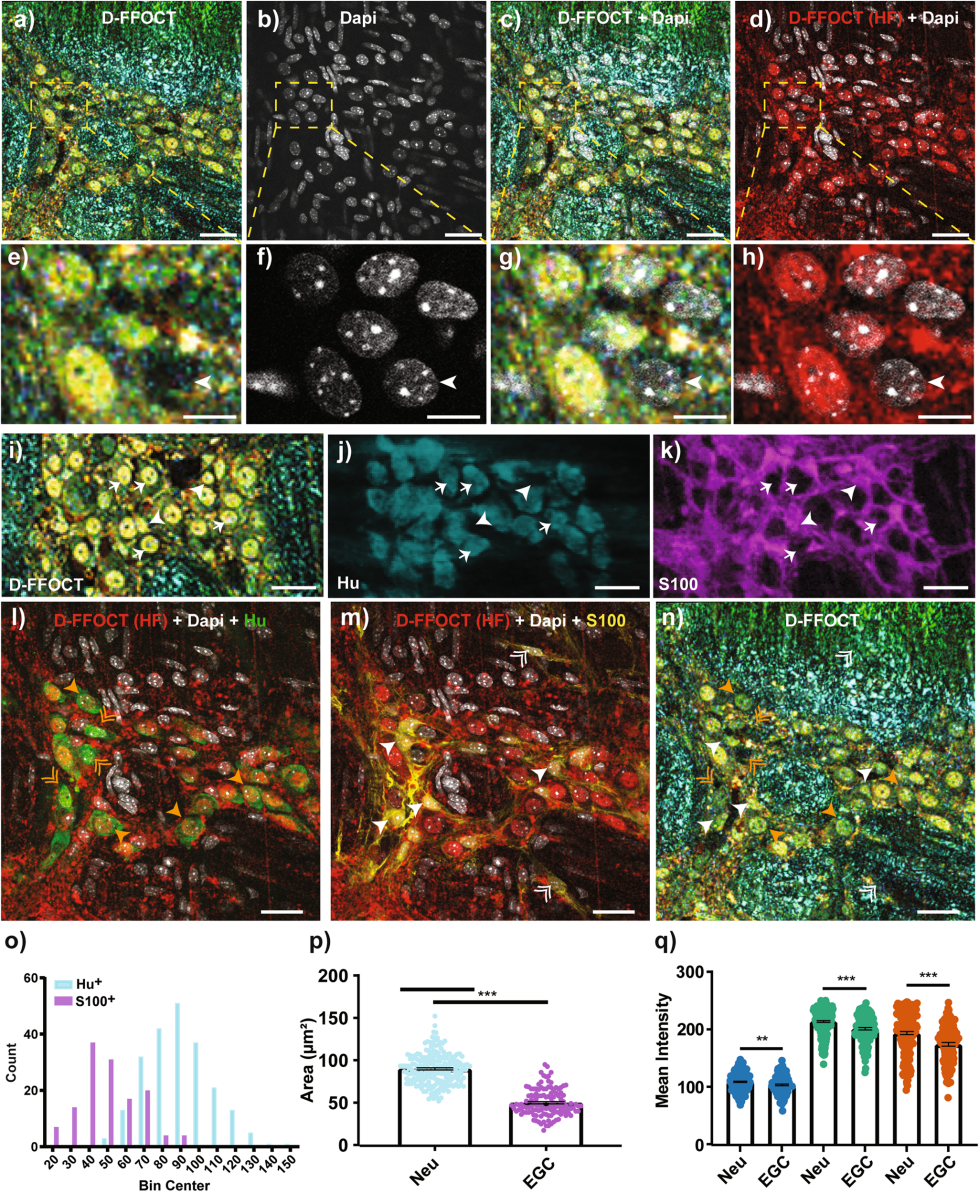
D-FFOCT enables the visualization of neuronal and glial nuclei in the mouse myenteric plexus. **a**–**d** Merged image (**c**, **d**) of D-FFOCT (**a**) and DAPI-stained confocal (**b**) micrographs. The same region of the mouse myenteric plexus was analyzed and compared. In **d**, only the high-frequency (red) image of D-FFOCT was superimposed with the DAPI confocal image (white) to clearly see signal colocalization. Scale bar: 25 µm. **e**–**h** enlargement of the dotted area in (**a**–**d**). Arrowhead points a DAPI-positive nucleus which is not detected by D-FFOCT. Scale bar: 10 µm. **i**–**k** Micrograph of the same ganglia visualized by D-FFOCT (**i**) or by apotome after Hu (**j**) or S100b (**k**) immunostaining. Hu and S100b cells overlapping with D-FFOCT positive-nuclei were pinpointed with arrows and arrowheads, respectively. Scale bar: 25 µm. **l**–**n** Merged image of high-frequency D-FFOCT (red), Dapi (white), and Hu (**l**; green) or S100b (**m**, yellow) confocal micrographs of a mouse myenteric ganglia. In the same ganglia, D-FFOCT-positive (simple arrowheads) and negative (double arrowheads) nuclei were indicated. Orange arrowheads and double arrowheads pointed D-FFOCT nuclei co-localized with Hu whereas white arrowhead and double arrowhead indicated D-FFOCT nuclei co-localized with S100b. Scale bar: 25 µm. **o** Size-frequency distribution of the D-FFOCT-positive nuclei which co-localized with Hu or S100b staining. 344 nuclei were analyzed within 13 ganglia from 3 mice (pooled in 10 µm^2^ range). **p** Mean area in µm^2^ of neuronal (Neu, *n* = 219) or glial (EGC; *n* = 125) D-FFOCT-positive nuclei. Mean value +/− SEM; Statistic: two-tailed Mann and Whitney t-test. **q** Mean intensity of neuronal (Neu, *n* = 219) or glial (EGC, *n* = 125)) nuclei for the low (blue), medium (bluish green), or high (vermillion) frequencies. Mean value +/− SEM; Statistic: two-tailed Mann and Whitney t-test. *p < 0.05; **p < 0.01, ***p < 0.001.

As a first approach to identify the structures detected by D-FFOCT in the myenteric plexus, D-FFOCT was combined with standard histological and immunostaining methods currently used to label nuclei and characterize cells of the enteric nervous system. For this purpose, tissue samples imaged by D-FFOCT were fixed and stained with 4’,6-diamidino-2-phénylindole (DAPI), a cell permeable fluorescent dye that binds to deoxyribonucleic acid (DNA). The superimposition of D-FFOCT images with high-power confocal micrographs of DAPI staining clearly showed that the nuclear-like structures detected in the myenteric plexus by D-FFOCT correspond to DAPI-labeled nuclei (Fig. 3a–h). Interestingly, not all nuclei could be visualized by D-FFOCT. Out of 261 DAPI-labeled nuclei counted in the myenteric ganglia, 27 were not visualized by D-FFOCT (white arrowhead in Fig. 3e–h). Of note, no D-FFOCT nucleus was detected in extra-ganglionic regions while cells like muscle cells could be present. This observation suggests cell- or morphology-dependent FFOCT signal.

To characterize cells with D-FFOCT-positive nuclei, the tissues were then fixed, immunostained using antibodies against neuronal (Hu) or glial (S100β) markers, and analyzed by apotome fluorescence microscopy. As illustrated in Fig. 3i–k, the great majority of the nuclear like structures observed by D-FFOCT overlapped with Hu (arrows) or S100β (arrow head) staining. The superimposition of D-FFOCT images to those obtained by confocal microscopy after immunohistochemistry and DAPI staining (Fig. 3l, m) revealed that D-FFOCT nuclear structures were detected in DAPI-positive Hu-positive cells (orange arrow heads in Fig. 3l, n). As indicated by orange double arrow heads, few DAPI-positive Hu-positive neurons did not exhibit D-FFOCT nuclear structures. Concerning the glial cells, D-FFOCT nuclear structures were detected in a great majority of DAPI-positive S100β-positive glia cells (white arrow heads in Fig. 3m, n) but some DAPI-positive S100β-positive cells were not visible by D-FFOCT (white double arrow heads in Fig. 3m, n). Cell counting showed that nuclear structures detected by D-FFOCT were present in 96% of neurons (163 nuclei out of 170) and 78% of EGC (71 nuclei out of 91) containing DAPI-positive nuclei.

To determine whether the morphology and/or the RGB intensity of the D-FFOCT nuclear structures could be used to discriminate nuclei from neurons and glial cells, their size distribution was analyzed. The results revealed Gaussian distributions. For glial cells, the peak was localized on the left part of the histogram which corresponded to the small nuclei while for neurons, the peak was more on the right which corresponded to the large nuclei (Fig. 3o). This observation is supported by the fact that the mean area of the EGC D-FFOCT nuclear structures was almost twice smaller than the mean area of the neuronal D-FFOCT nuclear structures (49.9 µm² +/− 15.8 vs 89.9 µm² +/− 17.9, respectively; mean +/− SD) (Fig. 3p). Interestingly, we showed that a cut-off value of 65 µm^2^, allowed us to discriminate 78.4% of the EGC and 92.7% of the neurons in the fractions below and over 65 µm^2^, respectively. Amplitudes of D-FFOCT frequencies were compared. The results indicated that the mean intensity of all frequencies signals was significantly larger in neurons as compared to glial cells (Fig. 3q).

### Pharmacological activation of neuronal activity modulates D-FFOCT signals in myenteric neurons and glial cells in situ

**Fig. 4:**
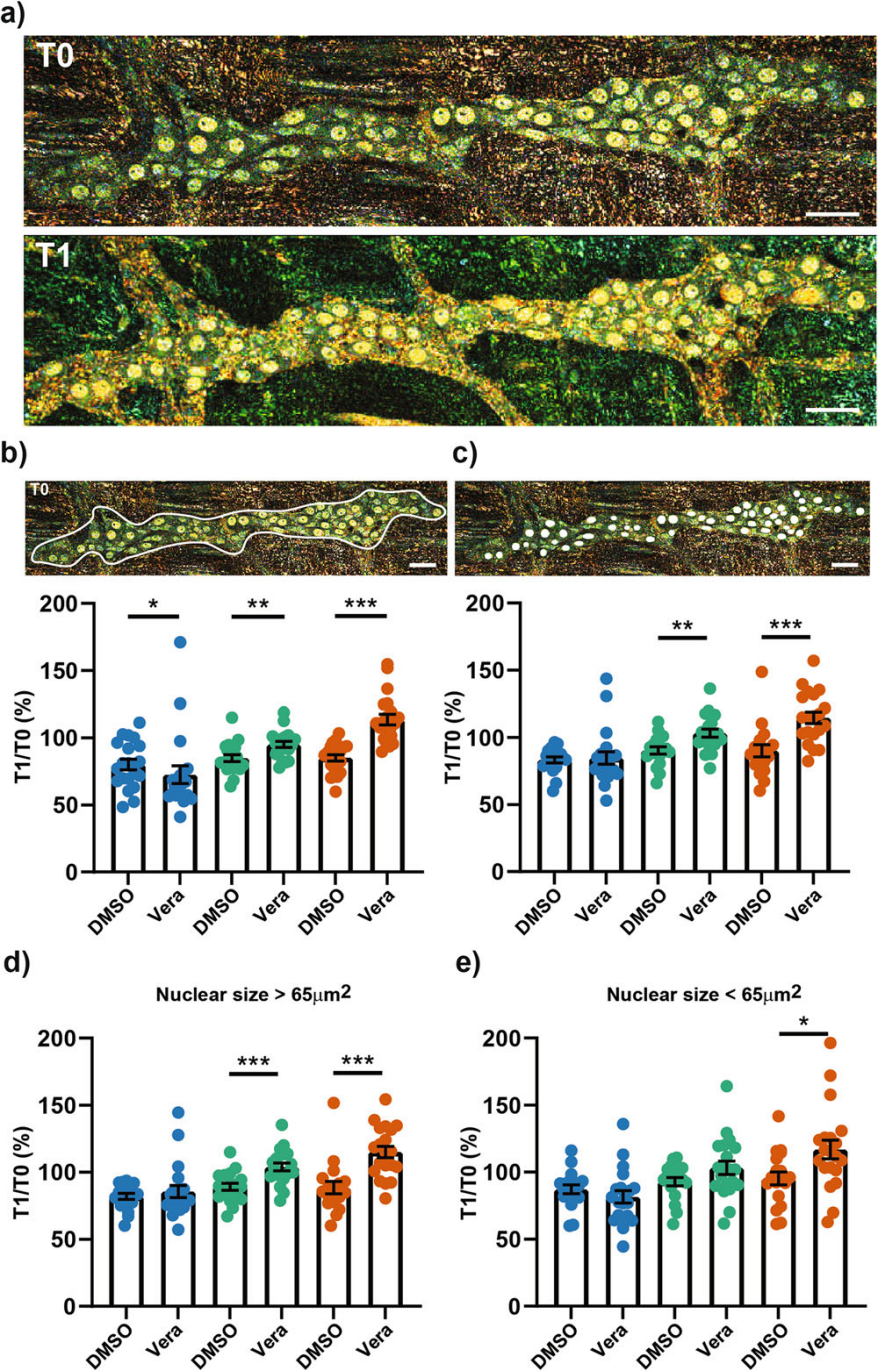
Effect of veratridine on D-FFOCT signal. **a** D-FFOCT micrograph of the same ganglia visualized before (T0) and after a 30 min treatment with 75 µM veratridine (T1). Scale bar: 30 µm. **b**, **c** T1:T0 ratio expressed as percentage of the mean intensity for low (blue), medium (bluish green), or high (vermillion) range of frequency after a treatment with vehicle (0.1% vol/vol DMSO) or 75 µM veratridine (Vera). Mean intensity was calculated from the whole ganglia (**b**) or from the nuclear structure (**c**) as indicated in the upper part of the panel. **d**, **e** Same experiment as in (**c**) but the nuclei were subdivided in function of their size >65 µm (**d**) and <65 µm (**e**). Veratridine, *n* = 20 ganglia from 4 mice; DMSO, *n* = 20 (**b**, **d**) or 18 (**c**, **e**) ganglia from 4 mice. Mean value +/− SEM; Statistic: two-tailed Mann and Whitney t-test. *p < 0.05; **p < 0.01, ***p < 0.001.

To determine whether D-FFOCT signals could be modulated by the neuronal activity, images of the whole ganglia were acquired by D-FFOCT before and 30 min after the addition of veratridine, a Na channel activator (Fig. 4a).

The mean intensity for low, medium, and high range of frequency was then analyzed. As observed in Fig. 4b, veratridine induced an increase in the intensity of high and medium-frequency signals and a significant reduction in the intensity of low-frequency signals in the whole ganglia as compared to control (Fig. 4b, 5 ganglia per animal per condition collected from 4 animals, control: DMSO 0.1% vol/vol). The impact of veratridine on D-FFOCT signals was then examined at a single nucleus level. As compared to control, an increase in the intensity of both medium and high-frequency signals was observed in veratridine-treated nuclei whereas no change was noticed for the low frequencies (Fig. 4c, 100–300 nuclei per animal in 4 animals). Interestingly, some D-FFOCT nuclear signals that were not detected under basal conditions, were observed in veratridine-treated nuclei (Supplementary Fig. 1a, arrows). To determine whether the changes in D-FFOCT signals were cell-type dependent, the results were classified as a function of the nuclear size cut-off value which discriminates between neuronal (nuclear size >65 µm^2^) and glial cells (nuclear size <65 µm^2^). As compared to control, veratridine treatment significantly increased the intensity of both medium and high-frequency signals in the cell population with nuclear size >65 µm^2^, which corresponded mainly to neurons (Fig. 4d). Veratridine also induced a significant increase in the intensity of high-frequency signals in the cell population with nucleus size <65 µm^2^, mainly constituted of glial cells (Fig. 4e). Finally, the changes in the D-FFOCT signals induced by veratridine were reversed following washout with Krebs solution (Supplementary Fig. 1b, c).

**Fig. 5:**
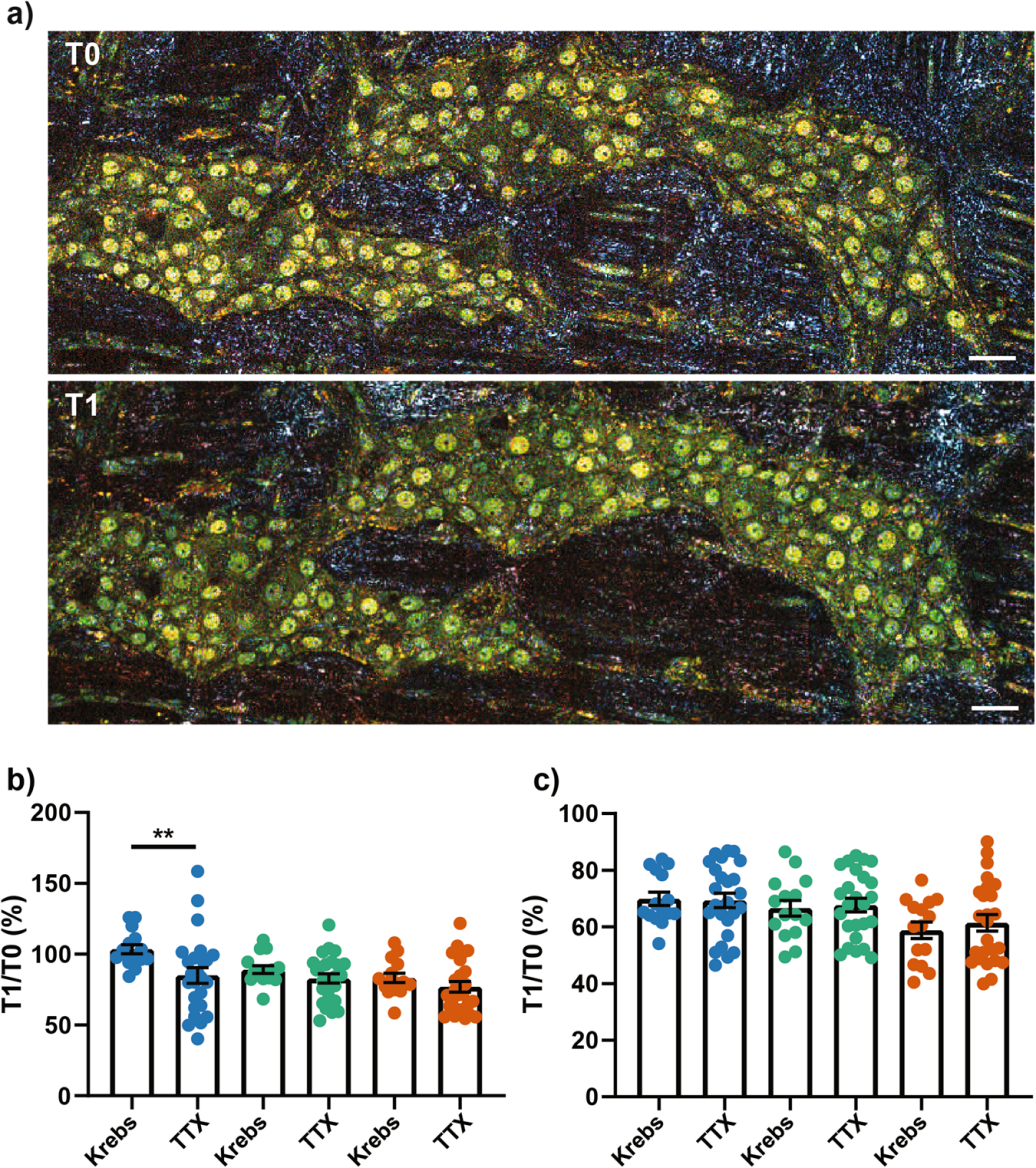
Effect of tetrodotoxin on D-FFOCT signal. **a** D-FFOCT micrograph of the same ganglia before (T0) and after a 30 min treatment with 1 µM of tetrodotoxin (T1). Scale bar: 30 µm. **b**, **c** Ratio T1:T0 in percentage of the mean intensity for low (blue), medium (bluish green), or high (vermillion) range of frequency for a treatment with vehicle (Krebs) or tetrodotoxin (TTX). Mean intensity was calculated from the whole ganglia (**b**) or from the nuclear structure (**c**). Tetrodotoxin, *n* = 25 ganglia from 5 mice; Krebs, *n* = 15 ganglia from three mice. Mean value +/−  SEM; Statistic: two-tailed Mann and Whitney t-test. **p < 0.01.

As the neuronal activity modulated the D-FFOCT signals in neuronal and glial cells, we examined whether pharmacological inhibition of the basal neuronal activity with the tetrodotoxin (TTX) impact the D-FFOCT signals (Fig. 5a). Analysis of the whole ganglia after treatment with TTX revealed that the toxin did not modify the intensity of medium or high-frequency signals but significantly reduced the intensity of low-frequency signals (Fig. 5b). Furthermore, no significant change in D-FFOCT frequency signals was observed in nuclear structures after treatment with TTX (Fig. 5c).

### Osmolarity modulates reversibly the D-FFOCT signals in myenteric neurons and glial cells in situ

Observations that increased neuronal activity modulated the D-FFOCT signals in both neuronal and glial nuclei prompted us to further investigate the putative mechanisms underlying these changes. Interestingly, it has been shown that neuronal activity is associated with modifications of the nuclear morphology due to changes in transcriptional activity^37^. Among other factors known to regulate the nuclear morphology is also osmotic stress^38,39^. In particular, hyperosmolarity has been shown to induce nuclear shrinkage or to contract nuclear morphology and, putatively, alter its vibrational properties whose modifications could be detected with D-FFOCT.

**Fig. 6:**
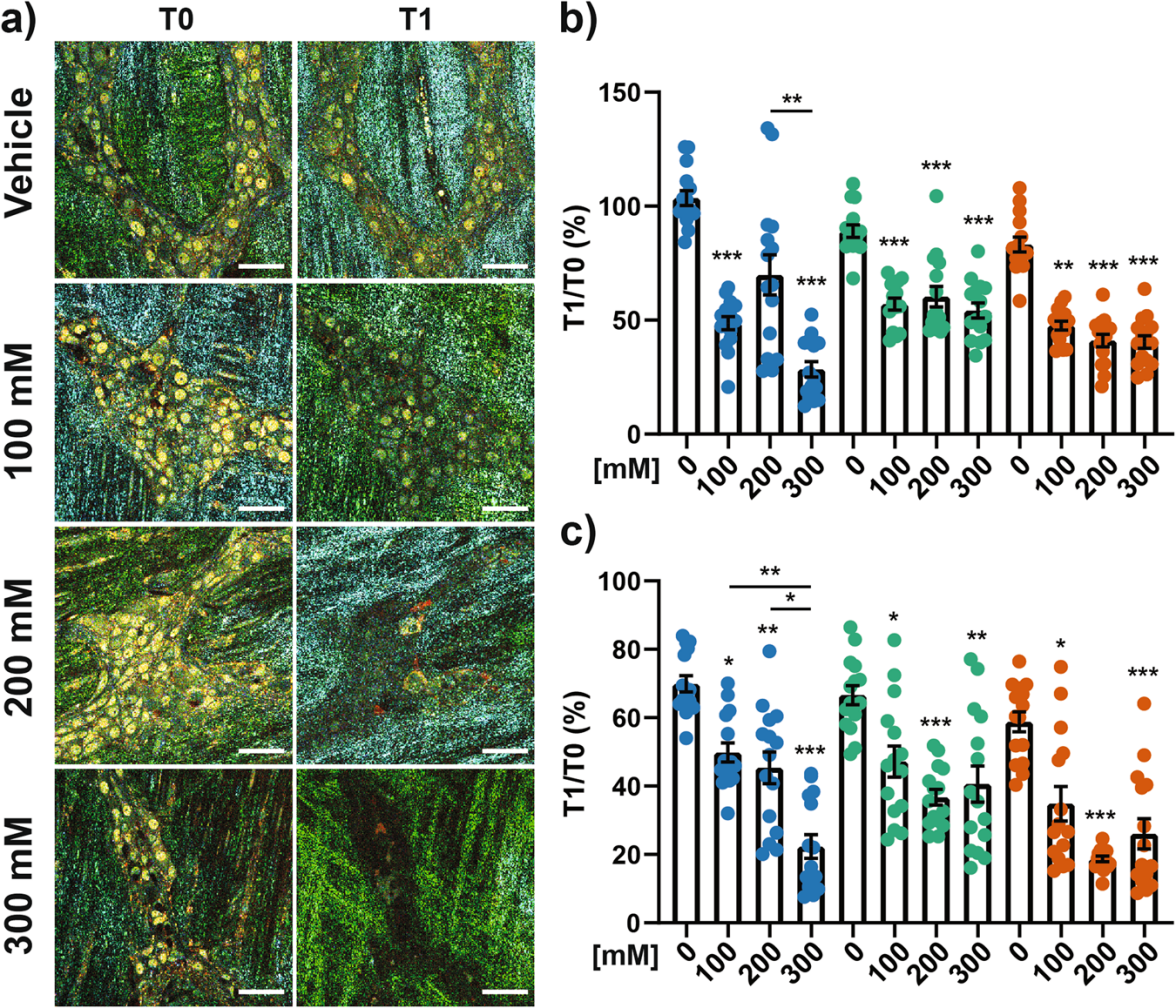
Dose–response effect of mannitol on D-FFOCT signal. **a** D-FFOCT micrograph of the same ganglia before (T0) and after a 30 min treatment with vehicle (Krebs), 100, 200, or 300 mM of mannitol (T1). Scale bar: 25 µm. **b**, **c** Ratio T1:T0 in percentage of the mean intensity for low (blue), medium (bluish green), or high (vermillion) range of frequency for a treatment with vehicle (Krebs, 0) or 100, 200, 300 mM of mannitol. Mean intensity was calculated from the whole ganglia (**b**) or from the nuclear structure (**c**). *n* = 15 ganglia from 3 mice. Mean value +/− SEM; Statistic: for each color, two-tailed Kruskal–Wallis test followed by a Dunn’s. *p < 0.05; **p < 0.01, ***p < 0.001.

To validate this hypothesis, we analyzed whether change in osmotic pressure had an impact on D-FFOCT signals in ENS cells (Fig. 6a). Interestingly, increasing concentration of mannitol induced a significant decrease in the intensity of D-FFOCT signals for low, medium and high frequencies in ganglia (Fig. 6b) but also in ganglionic cell nucleus (Fig. 6c). This effect was reversible since D-FFOCT signals in ganglia (Supplementary Fig. 2b) and in nuclei (Supplementary Fig. 2c) returned to their initial value after washout with Krebs solution (Supplementary Fig. 2a),

## Discussion

In the present study, we showed that time-frequency analysis of the OCT signals or D- FFOCT enabled in situ identification of enteric neurons and glial cells in preparations of mouse colon. Frequencies of OCT signals in nuclei of enteric neurons and glia were higher than those recorded in extra-ganglionic regions, and interestingly, external stimuli such as neuronal activation or hyperosmolarity induced reversible changes in OCT signal fluctuations. In the future, the use of this technique might bring novel insights into changes in enteric and glia functions in health and diseases.

The FFOCT imaging mode revealed low reflective structures at the interface between the longitudinal and circular muscles, which were identified by immunohistochemistry as being the myenteric plexus. Similar findings were previously obtained in mice using the OCT techniques, allowing the detection of changes in the density of myenteric ganglia in animal models of Hirschsprung’s disease^25^. FFOCT imaging also showed highly refractive structures matching the ganglia at the interface between the longitudinal muscles and the myenteric ganglia (Fig. 1a). These highly refractive structures are possibly formed by collagenous rich compounds, which molecules are important for the adhesion of the ganglia to the longitudinal muscles^40^. Interestingly, the composition of this extracellular matrix is altered in pathological conditions such as inflammatory bowel disease^41^ or Hirschsprung’s disease^42^. Therefore, FFOCT could be an interesting tool to detect changes in the composition of the extracellular matrix. To this regard, we recently showed a reduction in the high reflective FFOCT signals in the colonic mucosa of patients with spina bifida that were correlated with a significant decrease in the intensity of Sirius red staining, a marker of collagen deposits^43^.

FFOCT did not allow us to visualize individual cells in the myenteric ganglia, in contrast to other studies^25^. This discrepancy may be explained by technological differences such as objective lens or light source intensity between the commercial FFOCT system that we used (Light CT-scanner, LLTech), but D-FFOCT provides the possibility of imaging individual cells throughout the full thickness of the intestinal tissue, in particular in well-defined structures such as the myenteric plexus and the epithelial cell layer. Thus, this technical approach was recently used to discriminate epithelial cells in the human biopsies from the different parts of the intestinal tract^36^. In addition, D-FFOCT offers the possibility of integrating the subcellular movements and provides images according to the intensity of emitted frequencies. Subcellular components such as nuclei, characterized by elevated medium and high frequencies, were clearly visible by D-FFOCT in the myenteric ganglia, and immunohistochemistry analysis indicated that these nuclei corresponded to neurons or glial cells. Interestingly, 96% of the intraganglionic neurons were visible by D-FFOCT whereas few or no cellular elements were discerned by D-FFOCT in extra-ganglionic regions or in the muscular layers. As D-FFOCT signal might be linked to the metabolic activity of moving subcellular structures, it could be hypothesized that these differences are the consequence of different level of metabolic activity in the cells. Investigation of optimal frequency separation between three color channels could be interesting to further improve visualization of the ENS. In addition, some mismatching could be observed in the shape and positions of nuclei between D-FFOCT and confocal images after immunostaining. This is likely due to the deformations induced by the fixation and staining process between the two acquisitions, that we choose to neglect, and the registration error by itself which cannot be avoided due to the image resolution^44^. Indeed, registration was performed rigidly, with no deformation allowed, in order to avoid a biased warping of shapes. This rigid constraint allowed us to claim confidently that it was nuclei and not cells which can be observed in D-FFOCT, but led to the small mismatching observed.

In the present study, we also demonstrated that D-FFOCT signals in neurons and glial cells could be modified by external stimuli. We first showed that hyperosmolarity decreased FFOCT nuclear signals in a reversible manner. Interestingly, hyperosmolarity is known to alter the binding of protein to DNA and to induce a reversible DNA condensation process comparable to mitosis^45^. Therefore, the reversible decrease of D-FFOCT signals following the addition of increasing concentration of mannitol could be due to reduced DNA movement due to nucleus DNA condensation. Besides osmolarity, we aimed to determine the impact of neuronal activity on D-FFOCT signals as increased neuronal activity activates transcriptional activity in neurons, notably via intracellular pathways such mitogen-activated protein kinases^46^. Indeed, change in the activity-dependent chromatin states could have an impact on the vibrational state of the nucleus. Here, we reported that stimulation of the neuronal activity with veratridine triggered an increase in medium and high-frequency D-FFOCT signals in the nuclei of neurons and glial cells present in the myenteric ganglia. Although glial cells are not directly activated by veratridine, the increase in D-FFOCT signal could be the consequence of neuro-glia interactions following neuronal activation. Interestingly, nuclei that were not detected in the myenteric ganglia under basal conditions, were observed after treatment with veratridine (Supplementary Fig. 1). Such an induction was not observed following hyperosmolar stimuli. In addition, the reasons underlying the fact that no significant change in D-FFOCT frequency signals was observed in nuclear structures after treatment with TTX remain unknown. This might, however, be due to the fact that under basal condition neuronal activity is low so that addition of TTX would only induce small changes in activity that could not be detected with our system. Further investigations are required to understand the molecular basis of D-FFOCT signals in the myenteric ganglia but our data suggest that changes in the vibrational state of the nuclei could be linked to nuclear DNA condensation and/or variation in transcriptional activity.

Our present study provides the first demonstration that D-FFOCT could be used to image and identify in situ the neuronal and glial cells in the myenteric plexus. Changes in nuclei vibrational frequencies in response to physiological and non-physiological stimuli suggests that D-FFOCT could also be useful to analyze cell activity and detect abnormal cellular function.

## Methods

### Animals

Male C57BL/6J Rj mice aged 8–12 weeks (Janvier Laboratory, Le Genest-Saint-Isle, Fr) were housed in a 12-h light/dark cycle with ad libitum access to food and water. The investigators were accredited by the French National Veterinary Agency and the experiments were carried out in strict accordance with the recommendations of the local Animal Care and Use Committee of Nantes (France). After a 1-week adaptation period, animals were sacrificed by cervical dislocation for tissue collection. Whole intestine of each mouse was quickly excised and placed in an ice-cold Krebs solution (NaCl, 117 mM; KCl, 4.7 mM; MgCl_2_, 1.2 mM; NaH_2_PO_4_, 1.2 mM; NaHCO_3_, 25 mM; CaCl_2_, 2.5 mM; glucose, 11 mM).

### Tissue preparation

Intestinal tissues from adult mice were collected, washed with cold Krebs solution, and cut in small pieces. Then, segments of distal colon were opened longitudinally along the mesentery, stretched, and pinned on Sylgard (Dow Corning, Midland, MI) adapted for sample carrier.

### FFOCT and D-FFOCT imaging

FFOCT and D-FFOCT images were performed using the Light-CT scanner (LLtech, Paris, Fr) placed on an anti-vibratory table (CleanBench with Gimbal Piston vibration isolation, Photon Lines SAS, St Germain-en-Laye, Fr). The Sylgard carrier plate was placed in the scanner holder with sufficient solution to avoid drying out. After illuminating the sample with a halogen light source, the light reflected from the sample interfered with the reference arm light in Linnik interferometer, before being detected by a megapixel camera. Two-dimensional (2D) “en face” images were collected with a view field of view of 1.25 mm × 1.25 mm and voxel size of 1 µm × 1 µm × 1 µm. The system allowed the exploration of the sample depth up to around 150 µm in depth. For D-FFOCT, 1000 images at 300 Hz were collected (output interferometer images every 3.3 ms for 3.3 s). To ameliorate signal-to-noise performance, a non-overlapping average of four images was applied as a low pass filter to effectively dampen higher frequencies and in turn to suppress noise while measuring more photons. To maximize signal-to-noise ratio in the commercial Light-CT scanner the RGB integrating bands are pre-set to low-frequency blue channel (0–0.6 Hz), medium-frequency green channel (0.6–5.4 Hz), and high-frequency red channel (5.4–25 Hz).

### Immunohistochemistry

After FFOCT imaging, the colon samples were fixed in 4% paraformaldehyde in PBS for 3 h at room temperature, washed, and stored in PBS NaN_3_ (1 g per liter) until use. Permeabilization was achieved by incubating the tissues for 48 h–36 h in PBS NaN_3_ supplemented with 10% horse serum, 3% triton X100 and 0.05% saponin at 4 °C under agitation. Then, the samples were incubated for 24 h in permeabilized buffer with primary antibodies anti-Hu (human monoclonal antibody at 1:500, gift) and anti-S100b (rabbit polyclonal at 1:3, Dako Agilent, Santa Clara, USA) at room temperature under agitation. After washing, samples were incubated for 5 h in permeabilized buffer with secondary antibodies goat anti-rabbit Cy5 diluted at 1:500 (Jackson Immuno Research, Cambridge House, UK) and donkey anti-human Cy3 diluted at 1/500 (Jackson Immuno Research) at room temperature under agitation. After washing, nuclear counterstaining was performed for 10 min at room temperature, using DAPI (1:1000 in PBS Sigma-Aldrich, Saint Louis, USA) and samples were mounted between slide and cover slide with antifading mounting medium (Prolong gold antifade, Thermofisher, Waltham, USA).

### Microscopic imaging

Specimens were viewed using the fluorescent AxioZoom.V16 (Zeiss, Marly le Roi, Fr) associated with Apotome2. Confocal images were also obtained using the confocal microscope Nikon A1 RSi (Nikon SAS, Champigny sur Marne, Fr) with two objectives 60x and 20x. Images from FFOCT and apotome were aligned using the “Align Image by line ROI” plugin T. 3D D-FFOCT (Fiji software)^47^ while confocal 3D images were aligned using ICY software^48^ as described below.

### Superimposition of D-FFOCT and confocal images

Images of D-FFOCT were aligned using ec-clemv2^44^ under ICY.

In order to accurately assess the content of D-FFOCT at the cellular level, the registration was performed in 3D to take into account the sample change in pose between the two systems, and rigidly assuming that the tissue deformation will be negligible at this scale. After the D-FFOCT imaging (Supplementary Fig. 3a), the samples were imaged on the Nikon A1R confocal at 20x to obtain a reference image to ease the identification of the area of interest (Supplementary Fig. 3e). Then a stack on an arbitrary field of view was acquired at 60x (Supplementary Fig. 3f). The maximum intensity projection of the 20x was first manually registered to the D-FFOCT using structure of interest and setting the constraint to have no scaling or no deformation in the transformation (only rotation and translation allowed). This gives the 2D transformation matrix from 20x to D-FFOCT. Even if the number of pixels in both images was highly different, ec-clemv2 used the pixel size to compute the transformation in the micrometer unit, based on the images metadata given by the instruments allowing us to constrain the transformation to be rigid. The maximum intensity projection of the 60x confocal acquisition was automatically found in the 20x maximum intensity projection using the Autofinder part of ecclemv2. using a tailored icy protocol. This gives the 2D transformation from 60x to 20x. These transformations were combined in order to compute using the cascaded transformation function of ecclemv2 and was inversed to give the D-FFOCT to 60x 2D transformation. This transformation was applied to the D-FFOCT full stack, and served as initialization for the 3D rigid registration that was based on the manually picked center of what was hypothesized to match some nuclei (about 10 points localized in 3D in both images). The rigid constraint allowed us not to deform the images and to check the good matching of nuclei not used for registration.

### Analysis of neuronal and glial D-FFOCT nuclear size

To discriminate and quantify each nuclear size from neuronal and glial cells, D-FFOCT images and apotome images were superimposed with algorithm “Align Image by line ROI plugin” by FIJI.

For the nuclear analysis, masks were created from the D-FFOCT images using the WEKA plugin within Fiji. The masks were reported to split the red, green, and blue images. The 2D ROI, with parameters mean intensity, were then analyzed for each compartment. We determined mean value intensity of 125 glial nuclei and 219 neuron nuclei.

### Impact of veratridine or D-Mannitol on D-FFOCT signals

After marking the areas to be analyzed, each tissue sample pinned on Sylgard was imaged by D-FFOCT before treatment (T0). Sample was then removed from the carrier plate, placed in 6-well cell culture plates and treated with 75 µM of veratridine (Sigma-Aldrich, Saint Louis, USA), 1 µM of TTX (Bio-Techne SAS, Noyal-Châtillon sur Seiche, France), 100 mM, 200 mM, and 300 mM of D-Mannitol (Sigma-Aldrich), 0.1% (vol/vol) DMSO (veratridine control) or Krebs (TTX and Mannitol control) for 30 min at room temperature under agitation (200 rpm). Then, each treated-tissue sample pinned on Sylgard was returned to the scanner holder with the same pressure and localization (X, Y) to be imaged by D-FFOCT (T1). Each tissue sample was then washed twice 15 min each and imaged again by D-FFOCT (T2).

### Analysis of the D-FFOCT images

The D-FFOCT images of the ganglia acquired before treatment (T0), after chemical treatment (T1), and after the washing (T2) were aligned using the “Align function” within Fiji.

For the whole ganglia analysis, ganglia observed at T0 were manually surrounded to create a mask which was reported to split the red, green, and blue images acquired at T0, T1, and T2 and measure the mean intensity in the whole ganglia. Blue corresponds to low frequency (LF, 0–0.6 Hz), green to medium frequency (MF, 0.6–5.4 Hz), and red to high frequency (HF, 5.4–25 Hz).

For the nuclear analysis, masks were created from the images acquired at T0 using the WEKA plugin^49^ within Fiji (Supplementary Fig. 4). The masks were reported to split the red, green, and blue images. The mean intensity of each nucleus was calculated at T0, T1, and T2. When result were expressed in percent of T0, values were calculated for each nucleus and mean values per ganglia was used.

### Statistics and reproducibility

Number of independent experiments are specified in figure legends. Statistical significance was determined using the non-parametric two-tailed Mann and Whitney t-test or two-tailed Kruskal–Wallis test followed by a Dunn’s. All statistical analyses were performed using GraphPad Prism (GraphPad Prism 8.0, GraphPad software Inc) and differences with a *p*-value of 0.05 or less were considered statistically significant. *P < 0.05; **P < 0.01; ***P < 0.001.

### Reporting summary

Further information on research design is available in the Nature Portfolio Reporting Summary linked to this article.

## Supplementary information

Suplementary information

Description of Additional Supplementary Files

Supplementary Data.

Reporting Summary

## Data Availability

Numerical data generated or analyzed during this study are included in this published article (and its Supplementary data file). The micrographs presented in the current study are available in the [OMERO] repository [https://omero.os-bird.glicid.fr/webclient/?show=project-315 login: guest_user password: Welcome!1].
