# Supplementary material for: Visualizing enteric nervous system activity through dye-free dynamic full-field optical coherence tomography
Source: Commun Biol. 2023 Mar 2;6:236. doi: 10.1038/s42003-023-04593-9 (PMC9981581; doi:10.1038/s42003-023-04593-9)
Supplement: Supplementary file 1 — Suplementary information [file 42003_2023_4593_MOESM1_ESM.pdf]

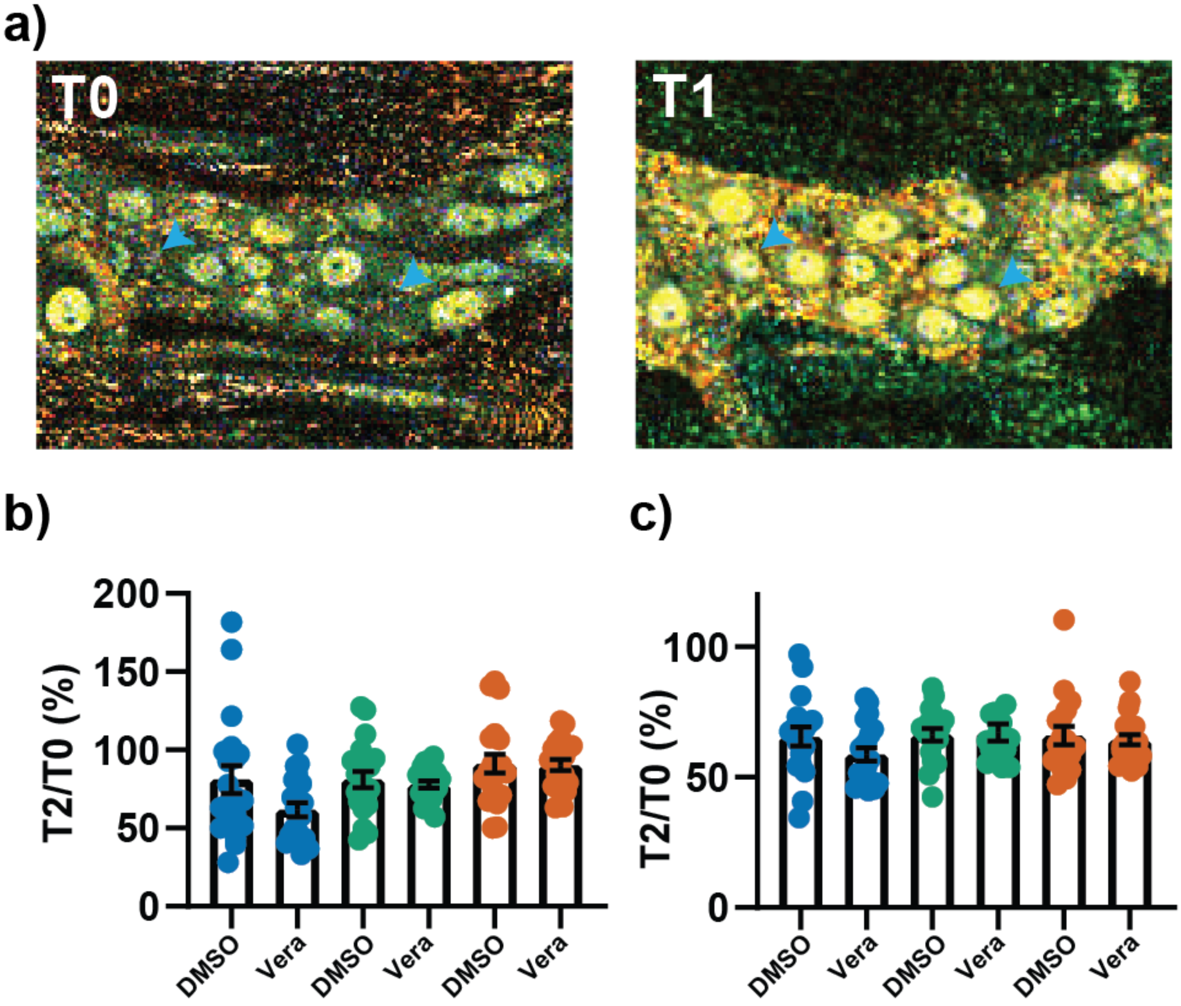


**Supplementary Figure 1**: **a**) D-FFOCT micrograph of the same ganglia before (T0) and after a 30 min treatment with 75 µM veratridine (T1). Arrows point D-FFOCT positive nuclei appearing post-treatment. Scale bar: 10 µm. **b-c**) Ratio T2/T0 in percentage of the mean intensity for (blue), medium (bluish green) or high (vermillion) range of frequency for a treatment of 30 min with vehicle (0.1% vol/vol DMSO) or 75 µm veratridine (Vera) followed by a wash of 30 min. Mean intensity was calculated from whole ganglia (b) or from the nuclear structure (c). Veratridine, n=20 ganglia from 4 mice; DMSO, n=20 (b) or 18 (c) ganglia from 4 mice. Mean value +/- SEM; Statistic: two-tailed Mann and Whitney t-test.

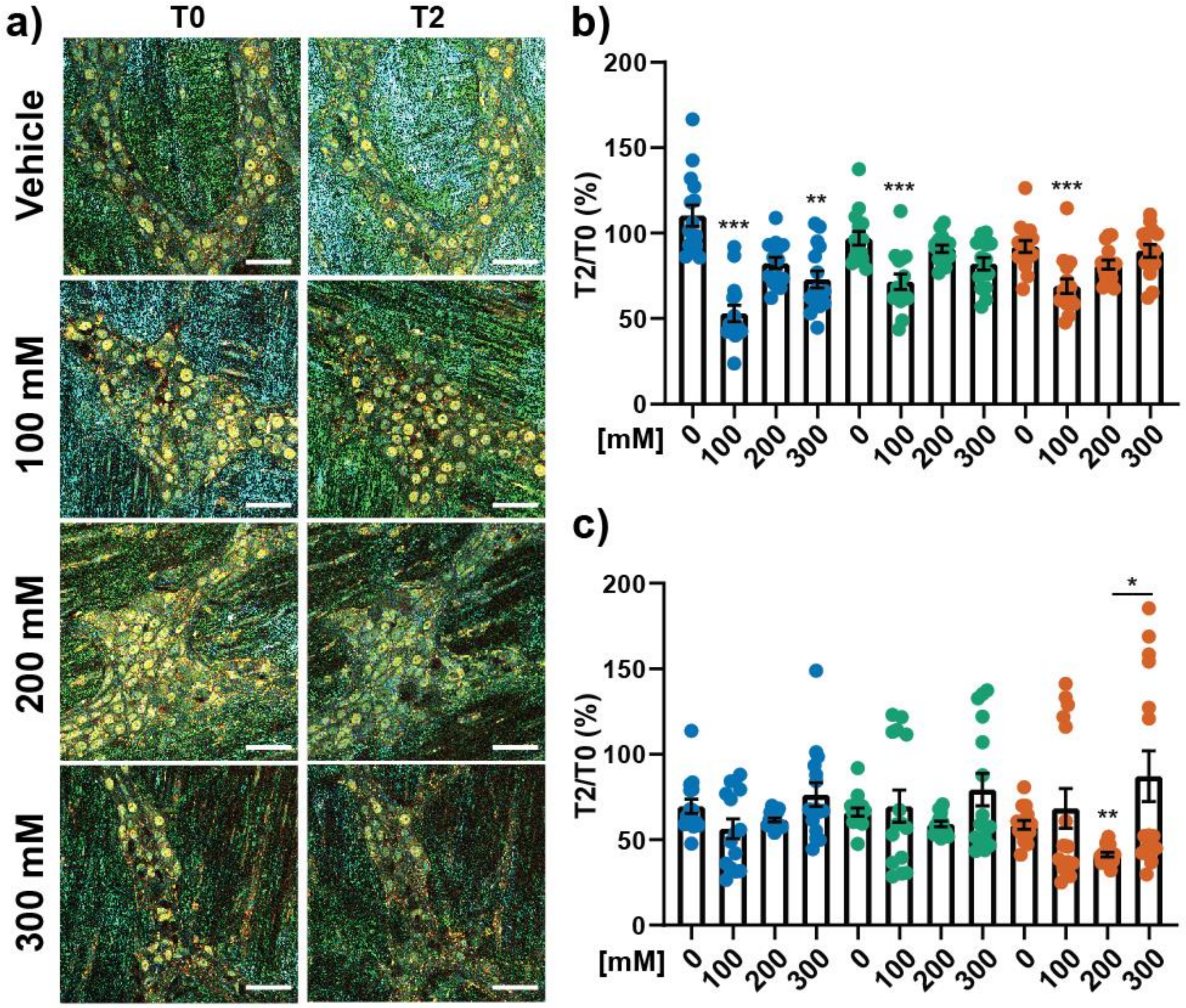


**Supplementary Figure 2**: **a**) D-FFOCT micrograph of the same ganglia before (T0) and after a 30 min treatment with vehicle (Krebs), 100, 200 or 300mM of mannitol followed by a 30 min post-treatment wash with Krebs (T2). Scale bar: 25 µm. **b-c**) Ratio T2/T0 in percentage of the mean intensity for (blue), medium (bluish green) or high (vermillion) range of frequency for a treatment with vehicle (Krebs, 0) or 100, 200, 300 mM of mannitol. Mean intensity was calculated from whole ganglia (b) or from the nuclear structure (c). n=15 ganglia from 3 mice. Statistic: for each color, two-tailed Kruskal-Wallis test followed by a Dunn's. *, p<0.05; **, p<0.01, ***, p<0.001.

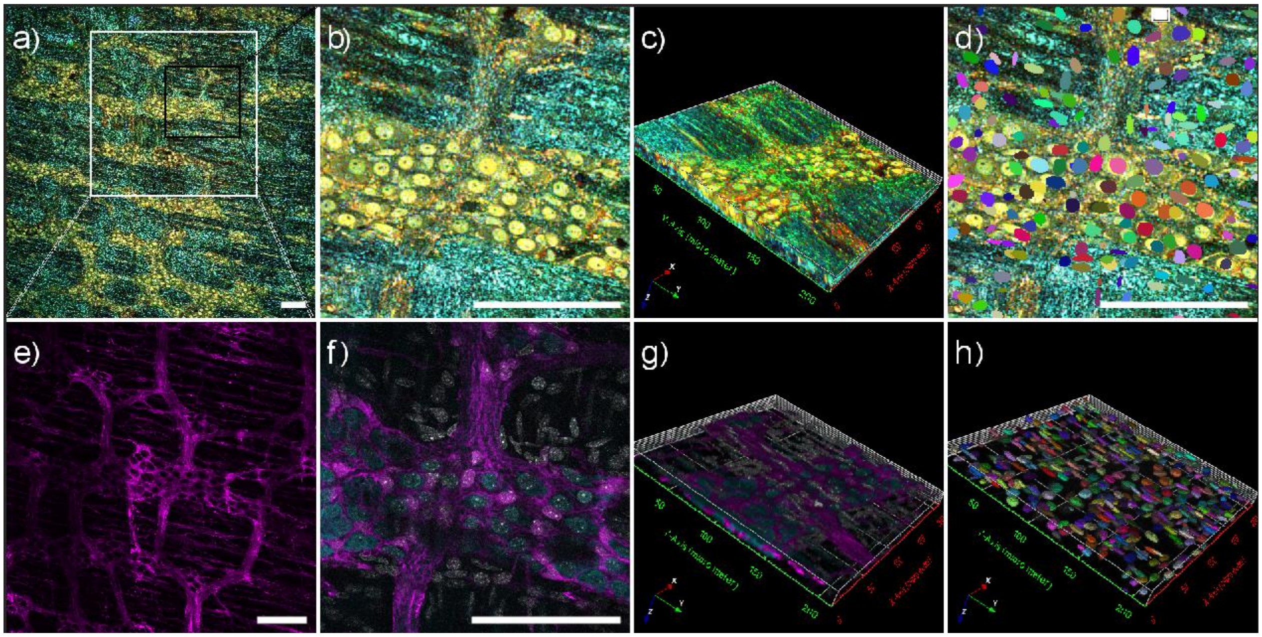


**Supplementary Figure 3**: **a)** D-FFOCT acquisition (slice 8 over 18). The white insert corresponds to the acquisition zone of the confocal image at 20x (e) and the black insert to the acquisition zone at 60x (f), to which it was rigidly registered in 3D (b). **b)** D-FFOCT acquisition 3D registered on the 60x confocal acquisition (slice 15 over 31 slices after registration). **c)** 3D rendering in ICY of the 3D registered D-FFOCT **d)** Same as (b) with nuclei segmented in 3D on the 60x confocal acquisition as colored overlays. **e)** Maximum intensity projection of the 20x confocal acquisition of the same sample, only channel S100b is displayed. **f)** Original 60 x confocal acquisition (slice 15 of 31) showing Hu in cyan, DAPI in grey, and S100b in magenta. **g)** 3D rendering in icy of the original 60x confocal stack. **h)** Same view as (g) but showing only the automatically segmented nuclei from DAPI signal. Scale bars are 100 µm.

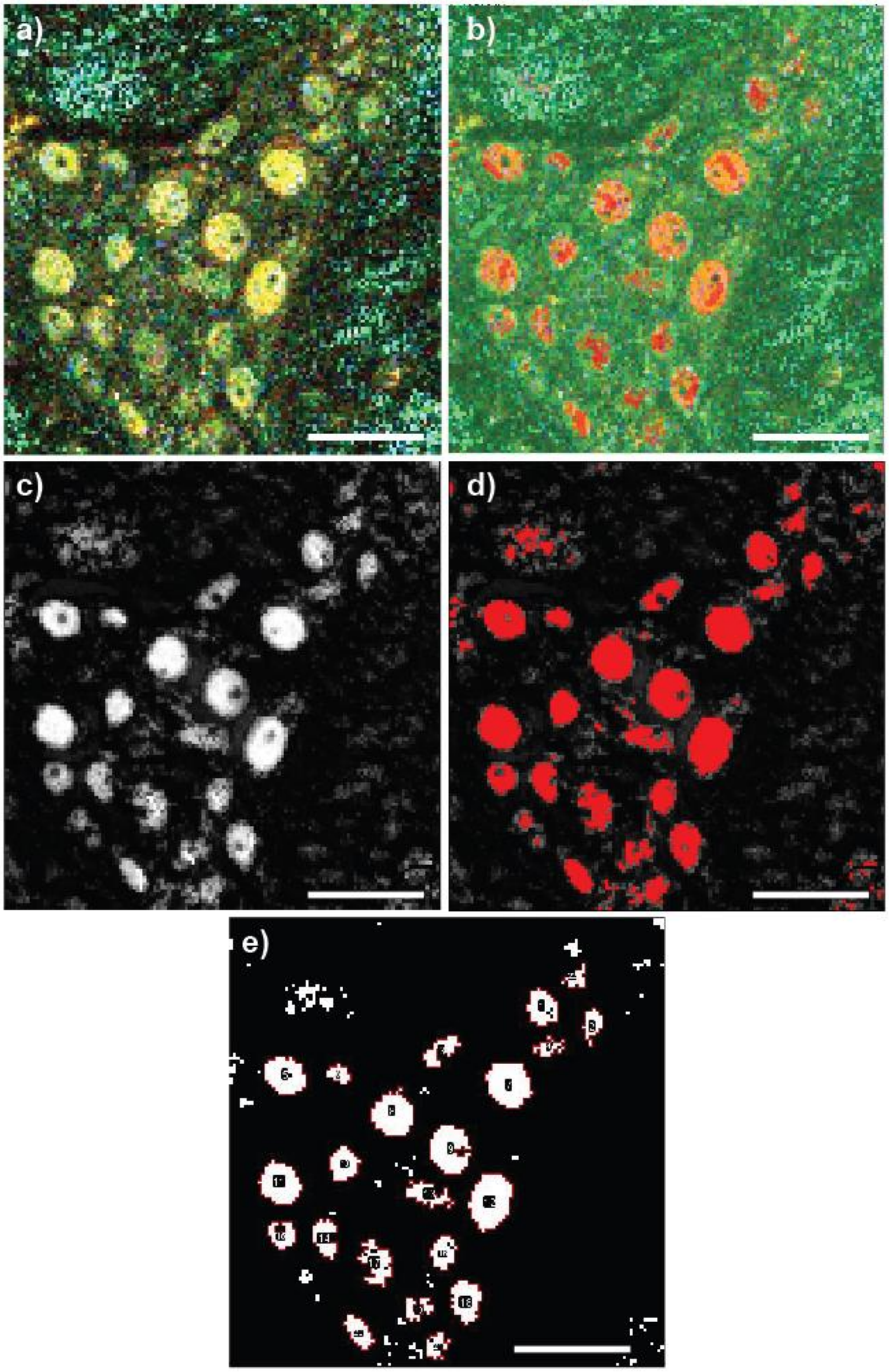


**Supplementary Figure 4**: ROI identification by weka procedure on D-FFOCT micrograph. **a)** D-FFOCT micrograph at the level of myenteric plexus. **b)** Trainable weka segmentation of the same region. **c)** Probability maps of the segmented region. **d)** Thresholding of micrograph c. **e)** Analysis particles of thresholded micrograph. Scale bars: a-e, 100 µm.
