# Supplementary material for: Visualizing enteric nervous system activity through dye-free dynamic full-field optical coherence tomography
Source: Commun Biol. 2023 Mar 2;6:236. doi: 10.1038/s42003-023-04593-9 (PMC9981581; doi:10.1038/s42003-023-04593-9)
Supplement: Supplementary file 2 — Description of Additional Supplementary Files [file 42003_2023_4593_MOESM2_ESM.pdf]

Description of Additional Supplementary Files

File name: Supplementary Data.

Description: Numerical data behind all the graphs in the paper. Data are identified in function of their figure number and panel letter.
